# Peritoneal Carcinomatosis in an Adult With Sjögren's Syndrome: A Diagnostic Dilemma Revealing a Rare Association With Primary Gastric Adenocarcinoma

**DOI:** 10.7759/cureus.8123

**Published:** 2020-05-14

**Authors:** Dawood Findakly

**Affiliations:** 1 Internal Medicine, Creighton University Arizona Health Education Alliance/Valleywise Health Medical Center, Phoenix, USA

**Keywords:** sjögren syndrome, autoimmune disease, peritoneal carcinomatosis, gastric adenocarcinoma, solid tumors

## Abstract

Sjögren's syndrome (SS) is a systemic autoimmune disease that mainly affects middle-aged women. It is rarely associated with solid neoplasms. We report a 60-year-old woman with a past medical history relevant for SS who was diagnosed with advanced-stage gastric adenocarcinoma upon evaluating for peritoneal carcinomatosis and succumbed two months after her original diagnosis. This case highlights the significance of considering gastrointestinal (GI) malignancy as an essential differential, particularly when evaluating patients with SS who fail conservative treatment for their GI symptoms.

## Introduction

Sjögren's syndrome (SS) is a systemic autoimmune condition with an average annual incidence of 4-6 per 100,000 population [1]. It occurs mainly among women within the fourth and sixth decades of life. SS manifests with a spectrum varying from mild sicca and dry mouth, characterized by mucosal dryness following exocrine glands infiltration by lymphocytes accompanied by B-cells activation, to severe systemic involvement [1-3].

In patients with SS, studies have illustrated a higher incidence of non-Hodgkin’s lymphoma (NHL) compared to the general population, but little is known about the risk of developing other cancer types, including solid tumors [4,5].

This rare case report describes a 60-year-old woman with a history of SS who presented with abdominal distension, found to have ascites from peritoneal carcinomatosis, and eventually diagnosed with poorly differentiated gastric adenocarcinoma.

## Case presentation

A 60-year-old woman with a past medical history pertinent for hypothyroidism and SS presented to the ED complaining of worsening abdominal distention that started six weeks prior. Of note, the patient was evaluated and treated in the past several months with a proton pump inhibitor (PPI), omeprazole, for suspected gastritis by her primary care physician (PCP). The patient, however, was having persistent symptoms despite the treatment and was sent to a gastrointestinal (GI) physician for further evaluation. Unfortunately, she was unable to see a GI physician for several months and could not take the discomfort any longer, and thus, her PCP sent her to the ED. Upon her ED presentation, she relates that her condition was associated with fatigue, nausea, anorexia, and denied any fever, chills, night sweats, change in weight, or bowel habits. She reports only taking levothyroxine and occasional diclofenac sodium. She denied any family history of cancers. She had an unremarkable screening mammogram and pap smear that was done 12 months ago. She denied any herbal medication use, exposure to chemicals, tobacco, alcohol, or illicit drug use.

**Figure 1:**
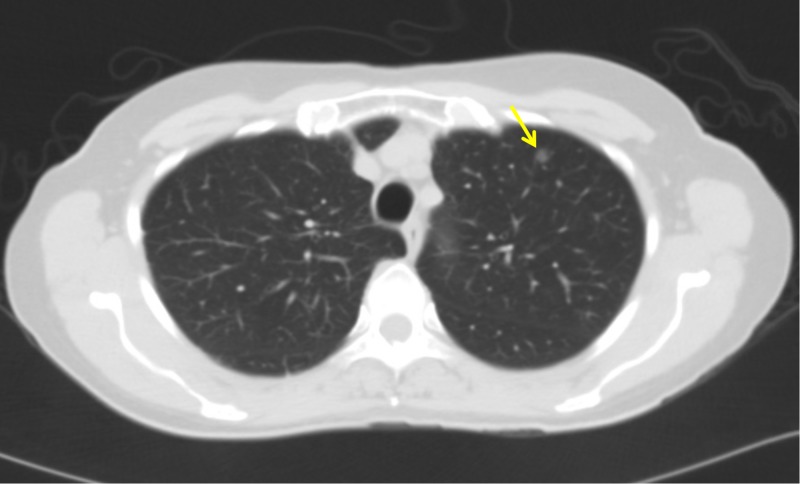
CT scan of the chest demonstrating a 6 mm left upper lobe pulmonary nodule (arrow)

**Figure 2:**
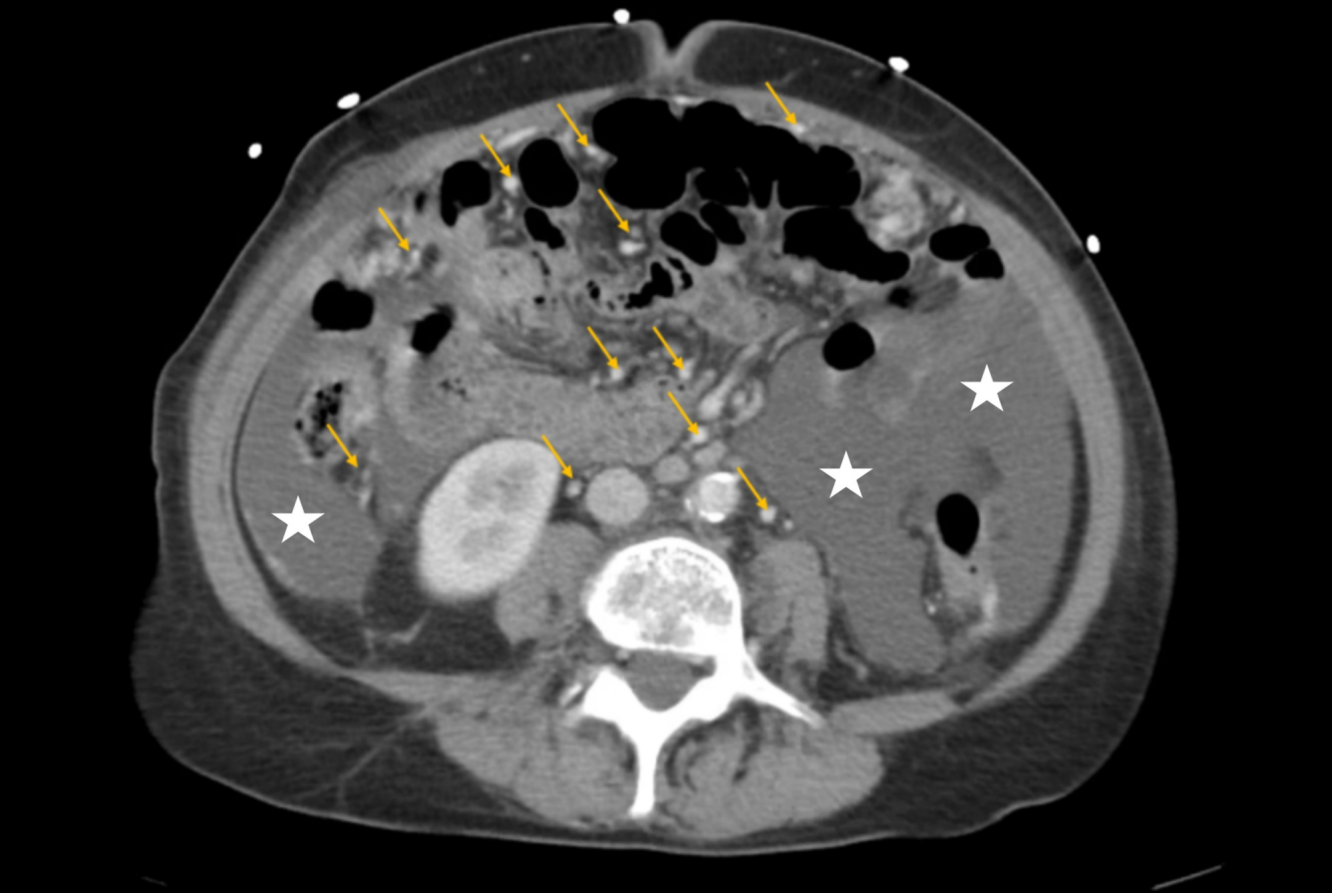
CT scan of the abdomen and pelvis showing ascites (white stars) with mesenteric, and retroperitoneal lymphadenopathy (yellow arrows)

The physical examination was pertinent for a distended, non-tender abdomen. Laboratory workup was relevant for white blood cell (WBC) of 6.2 x 10^3^/µL, hemoglobin of 11/8 g/dL, platelet count of 300 K/µL, sodium of 140 mmol/L, potassium of 4 mmol/L, creatinine of 0.9 mg/dL, lactic acid of 1.2 mmol/L, prothrombin time of 11.2 seconds, and an INR of 1.1. CT scan of the chest showed 6 mm left upper lobe and 1 cm right middle lobe pulmonary nodules (Figure 1). CT scan of the abdomen and pelvis revealed a large amount of ascitic fluid, with diffuse omental thickening, extensive mesenteric, and retroperitoneal lymphadenopathy (Figure 2). These findings suggested a neoplastic process secondary to peritoneal carcinomatosis.

**Figure 3:**
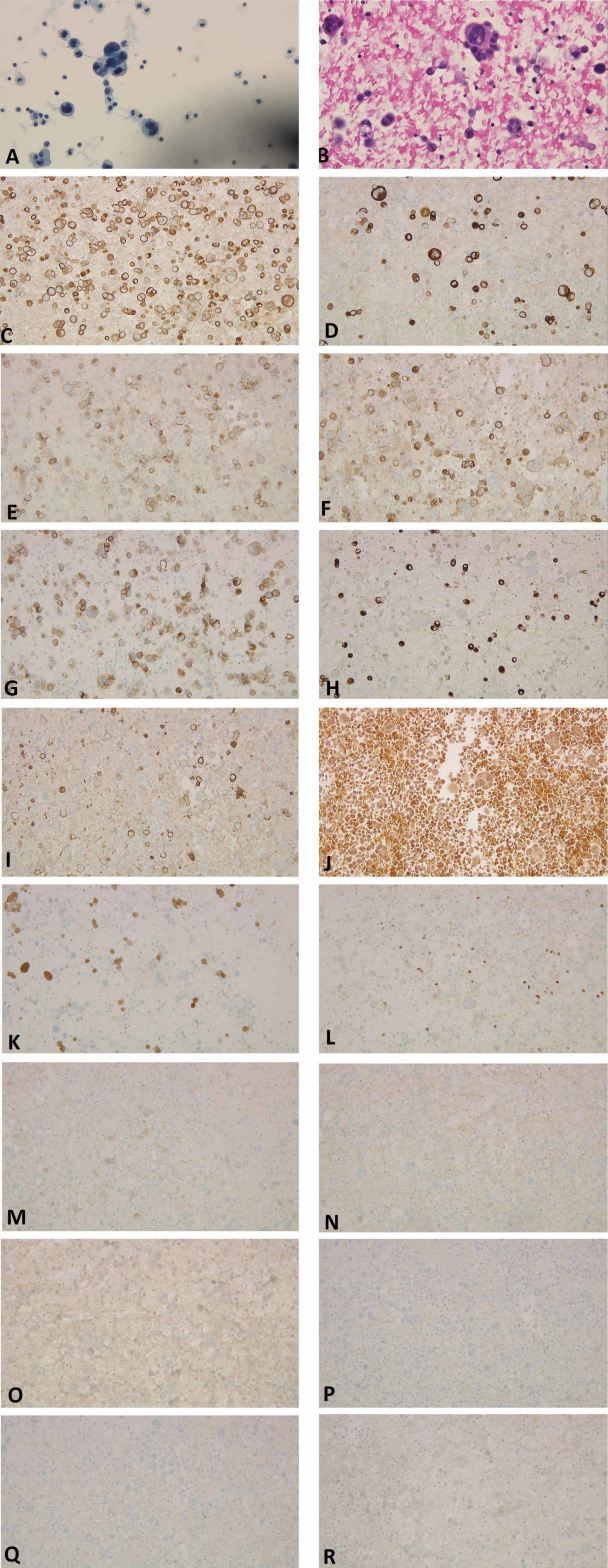
Microscopic peritoneal fluid cytology showing malignant cells with enlarged nuclei and nucleoli. A: thin preparation at 400x magnification; B: cell clock preparation at 400x magnification. Background of neoplastic cells (at 200x magnification) positive for C: CK19; D: CK20; E: BerEP4; F: CEA; G: MOC-31. Rare tumor cells positive for H: CK7; and I: HBME-1. Cells are negative for J: mucicarmine; K: calretinin; L: WT-1; M: ER; N: mammoglobin; O: TTF-1; P: caldesmon; Q: D2-40; and R: PAX-8 CK: cytokeratin; CEA: carcinoembryonic antigen; HBME-1: human bone marrow endothelium marker-1; WT-1: Wilms tumor protein 1; ER: estrogen receptor; TTF-1: thyroid transcription factor 1; D2-40: podoplanin; PAX-8: paired box gene 8

Thereafter, the patient had a diagnostic and therapeutic paracentesis. The ascitic fluid SAAG was 1.1, and cytologic examination revealed numerous metastatic cells positive for CK19, CK20, BerEP4, CEA, and MOC-31. Rare cells were positive for CK7 and HBME-1 and were negative for mucicarmine, calretinin, WT-1, ER, mammaglobin, TTF-1, caldesmon, D2-40, and PAX-8. There was no definitive staining for CA-125 (Figure 3A-R). These findings were consistent with poorly differentiated carcinoma of unknown primary. Further testing revealed CA125 was elevated at 434 U/mL.

**Figure 4:**
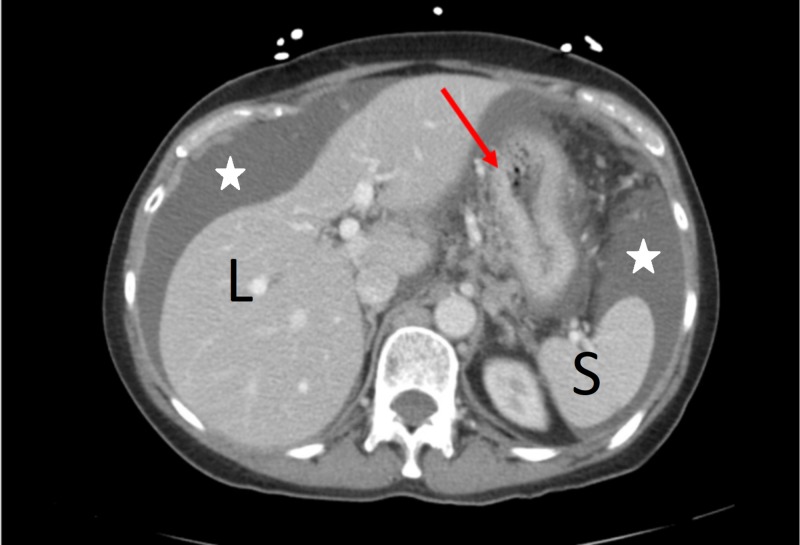
CT scan of the abdomen showing gastric wall thickening (red arrow) and ascites (white stars) L: liver; S: spleen

Given that the ovaries were small with no apparent masses, and the uterus was normal-appearing, therefore, the radiologists were asked to review the CT scan images where they noticed that the stomach wall appeared to be abnormally thickened, even despite being under-filled (Figure 4). Therefore, the gastric source of her cancer was placed higher on the differential, especially with her upper GI symptoms that were not responsive to PPI therapy. GI physician evaluated the patient, and thus, endoscopy with esophagogastroduodenoscopy (EGD) and colonoscopy was then advised as the gastric malignancy was in the differential. EGD showed a circumferential malignant appearing mass lesion in the gastric fundus, which was biopsied, and colon was unremarkable. Pathology revealed poorly differentiated adenocarcinoma with surface ulceration of the gastric fundus, and the patient was diagnosed with stage IV gastric adenocarcinoma.

The patient was later discharged from the hospital and referred to the oncology clinic. The patient was offered palliative chemotherapy with IV cisplatin and oral capecitabine. However, she can't swallow pills, and thus, she was offered the option of carboplatin with paclitaxel, or carboplatin with 5-FU (fluorouracil) but the patient felt that she was too weak and fatigued to tolerate the therapy and thus, she elected to not pursue palliative chemotherapy and desired hospice therapy for comfort care. The patient died two months after her initial diagnosis.

## Discussion

Globally, gastric carcinoma endures one of the most common cancers, with approximately 27,510 patients are diagnosed annually in the United States, of whom 11,140 are expected to die [6]. Gastric adenocarcinoma is the most common type of primary gastric carcinoma and accounts for 95% of cases, followed by primary gastric lymphoma [7].

Malignancy was reported as the leading cause of death in patients with SS [8]. The relation of autoimmune diseases with increased risk of malignancy has been well established. In particular, the increased risk of NHL in SS patients is widely recognized, though the uncertainty regarding the risk of cancers other than NHL in these patients remains obscure [4,5,9]. Extra-nodal sites of involvement by NHL could also be present, with the stomach being seldom reported in the literature [10]. Moreover, the SS association with an increased incidence of lymphoma, multiple myeloma, breast, and thyroid carcinoma with respect to the normal population was reported in the literature [1,11].

Zhang et al. reported 29 patients (2.2%) to develop malignancies out of 1,320 patients with SS upon follow-up. Out of those 29 patients, 19 patients (65.5%) developed solid malignancies, and only two patients (6.9%) developed gastric adenocarcinoma [12]. Therefore, making our patient the third published case that addresses the association between gastric adenocarcinoma in patients with SS and the first published case report detailing the patient’s clinical course, management, and outcome.

This report details the unique presentation of gastric adenocarcinoma. Our patient had non-specific GI symptoms that were not responsive to PPI therapy for several months before presenting with malignant ascites. In our patient, a meticulous search for clues for the primary location of the tumor through evaluating the patient’s history combined with simultaneously re-evaluating the CT scan images from prior studies guided the diagnosis of gastric adenocarcinoma. This diagnosis was established with histologic evidence through endoscopic tissue biopsy.

## Conclusions

This article verifies the association between SS and gastric adenocarcinoma and highlights the importance of early diagnosis as delay in diagnosis leads to poor outcomes. The rare association of SS with metastatic gastric carcinoma should be in the differential for patients with SS who presents with GI symptoms and failed initial conservative therapy. Further studies are required to determine the true incidence and convey clinical expertise in establishing guidelines for solid tumors in patients with autoimmune disorders, which could, therefore, aid in improving outcomes.

## References

[1] Weng MY, Huang YT, Liu MF, Lu TH (2012). Incidence of cancer in a nationwide population cohort of 7852 patients with primary Sjogren's syndrome in Taiwan. Ann Rheum Dis.

[2] Ramos-Casals M, Brito-Zerón P, Sisó-Almirall A, Bosch X (2012). Primary Sjogren syndrome. BMJ.

[3] Lai WS, Liu FC, Wang CH, Chen HC (2014). Unusual cancer in primary Sjögren syndrome. Can Fam Physician.

[4] Brito-Zerón P, Kostov B, Fraile G (2017). Characterization and risk estimate of cancer in patients with primary Sjögren syndrome. J Hematol Oncol.

[5] Anderson LA, Gadalla S, Morton LM (2009). Population-based study of autoimmune conditions and the risk of specific lymphoid malignancies. Int J Cancer.

[6] Siegel RL, Miller KD, Jemal A (2019). Cancer statistics, 2019. CA Cancer J Clin.

[7] Rawla P, Barsouk A (2019). Epidemiology of gastric cancer: global trends, risk factors and prevention. Prz Gastroenterol.

[8] Lorenzo R, Argibay A, Sousa A (2016). Sjögren syndrome, cancer incidence and mortality in Vigo area. Ann Rheum Dis.

[9] Liang Y, Yang Z, Qin B, Zhong R (2014). Primary Sjogren's syndrome and malignancy risk: a systematic review and meta-analysis. Ann Rheum Dis.

[10] Voulgarelis M, Dafni UG, Isenberg DA, Moutsopoulos HM (1999). Malignant lymphoma in primary Sjögren's syndrome: a multicenter, retrospective, clinical study by the European Concerted Action on Sjögren's Syndrome. Arthritis Rheum.

[11] Brown A, Luckhardt TR (2014). Adenocarcinoma of the lung and Sjogren's syndrome: an unlikely pair. Am J Respir Crit Care Med.

[12] Zhang W, Feng S, Yan S (2010). Incidence of malignancy in primary Sjogren's syndrome in a Chinese cohort. Rheumatology (Oxford).

